# PTGES Expression Is Associated with Metabolic and Immune Reprogramming in Pancreatic Ductal Adenocarcinoma

**DOI:** 10.3390/ijms24087304

**Published:** 2023-04-15

**Authors:** Divya Murthy, Kuldeep S. Attri

**Affiliations:** 1 Eppley Institute for Research in Cancer and Allied Diseases, University of Nebraska Medical Center, Omaha, NE 68198, USA; 2 Department of Molecular and Human Genetics, Baylor College of Medicine, Houston, TX 77030, USA

**Keywords:** pancreatic cancer, PTGES, metabolism, glycolysis, immune regulation

## Abstract

Metabolic reprogramming is an established hallmark of multiple cancers, including pancreatic cancer. Dysregulated metabolism is utilized by cancer cells for tumor progression, metastasis, immune microenvironment remodeling, and therapeutic resistance. Prostaglandin metabolites have been shown to be critical for inflammation and tumorigenesis. While the functional role of prostaglandin E2 metabolite has been extensively studied, there is a limited understanding of the PTGES enzyme in pancreatic cancer. Here, we investigated the relationship between expression of prostaglandin E synthase (PTGES) isoforms and the pathogenesis and regulation of pancreatic cancer. Our analysis identified higher expression of PTGES in pancreatic tumors compared to normal pancreatic tissues, suggesting an oncogenic function. Only PTGES1 expression was significantly correlated with worse prognosis of pancreatic cancer patients. Further, utilizing cancer genome atlas data, PTGES was found to be positively correlated with epithelial-mesenchymal transition, metabolic pathways, mucin oncogenic proteins, and immune pathways in cancer cells. PTGES expression was also correlated with higher mutational burden in key driver genes, such as TP53 and KRAS. Furthermore, our analysis indicated that the oncogenic pathway controlled by PTGES1 could be regulated via DNA methylation-dependent epigenetic mechanisms. Notably, the glycolysis pathway was positively correlated with PTGES and may fuel cancer cell growth. PTGES expression was also associated with downregulation of the MHC pathway and negatively correlated with CD8+ T cell activation markers. In summary, our study established an association of PTGES expression with pancreatic cancer metabolism and the immune microenvironment.

## 1. Introduction

Pancreatic cancer is the fourth leading cause of cancer-related deaths in the United States, having a poor prognosis. The median survival is merely six months, and the five-year survival rate of pancreatic ductal adenocarcinoma (PDAC) patients is a dismal 11% [1]. Due to the lack of improved therapeutic outcomes, PDAC is predicted to become the second leading cause of cancer mortality by 2030 [2]. The poor prognosis of pancreatic cancer can be attributed to various factors, including late diagnosis, absence of specific biomarkers, and resistance to currently available targeted therapies [3]. In addition, large-scale genomic studies have revealed genetic alterations in the KRAS, TP53, SMAD4, and CDKN2A genes as key drivers of PDAC progression [4,5,6,7]. KRAS mutations, observed in more than 90% of pancreatic cancer patients, initiate tumorigenesis and drive neoplastic progression [8]. Altered KRAS signaling results in regulation of phospho-signaling pathways, the fibro-inflammatory microenvironment, immune cell remodeling, autophagic induction, and cell-intrinsic metabolic rewiring [9,10]. While therapeutic efforts to inhibit KRAS have failed in the past, there is renewed hope with KRAS G12C- and KRAS G12D-specific inhibitors [8,11,12]. Since these new inhibitors are still in the initial phase of development, identifying and targeting downstream metabolic nodes have emerged as attractive approaches for novel PDAC therapy.

The PDAC tumor microenvironment (TME) is highly complex, with augmented metabolic interactions among cancer, stromal, and immune cells that facilitate tumor progression [13,14,15]. Interestingly, these metabolic changes are closely associated with molecular abnormalities in PDAC, including hyperactivated growth factor signaling, epigenetic regulation, dysregulated gene expression, and abnormal posttranslational modifications [16]. A principal barrier to pancreatic cancer treatment is the dense fibrotic stroma and hypoxic microenvironment. Due to the reduced vasculature, pancreatic cancer cells depend on dysregulated metabolic programming to convene their energetic and biosynthetic demands [17,18]. Pancreatic cancer cells exhibit broad metabolic plasticity and utilize distinct substrates via the Warburg effect, reverse Warburg effect, glutaminolysis, and lipid-dependent carbon sources. Additionally, symbiotic metabolic interactions between cancer and other cells of the TME support pancreatic cancer metabolism and growth. The metabolites directly exchanged between cancer and immune cells can trigger signaling pathways, resulting in impairment of antitumor immunity [19].

Prostaglandins (PGs) and other eicosanoids are bioactive lipids that influence cancer progression by altering the adhesive, migratory, and invasive behavior of cells [20]. Biochemically, PGs are derived by cyclooxygenase (COX)-mediated conversion of the 20-carbon chain fatty acid arachidonic acid into an endoperoxide intermediate that is further metabolized to PGs, such as PGE2, PGD2, PGF2α, prostacyclin (PGI2), and thromboxane A2, through PG synthases [21]. The resulting PGE2 binds to and activates G-protein-coupled prostaglandin receptors that further mediate interactions with the ECM through Src and focal adhesion kinase (FAK) [20]. PGE2 triggers inflammation, angiogenesis, and metastasis and suppresses antitumor immunity. Moreover, inhibition of PGE2 secretion by COX inhibitors is associated with reduced risk of multiple cancers, including breast, prostate, lung, ovarian, and gastric cancers [22]. Prostaglandin pathway metabolites, including PGE2, were elevated in pancreatic cancer tissues and associated with cancer progression [19]. In this study, we evaluated the prognostic significance and function of PGE2 synthesizing PTGES isoforms in regulating multiple aspects of pancreatic cancer progression using meta-analysis of publicly available datasets.

## 2. Results

### 2.1. PTGES Expression Is Increased in PDAC Patients

**Figure 1:**
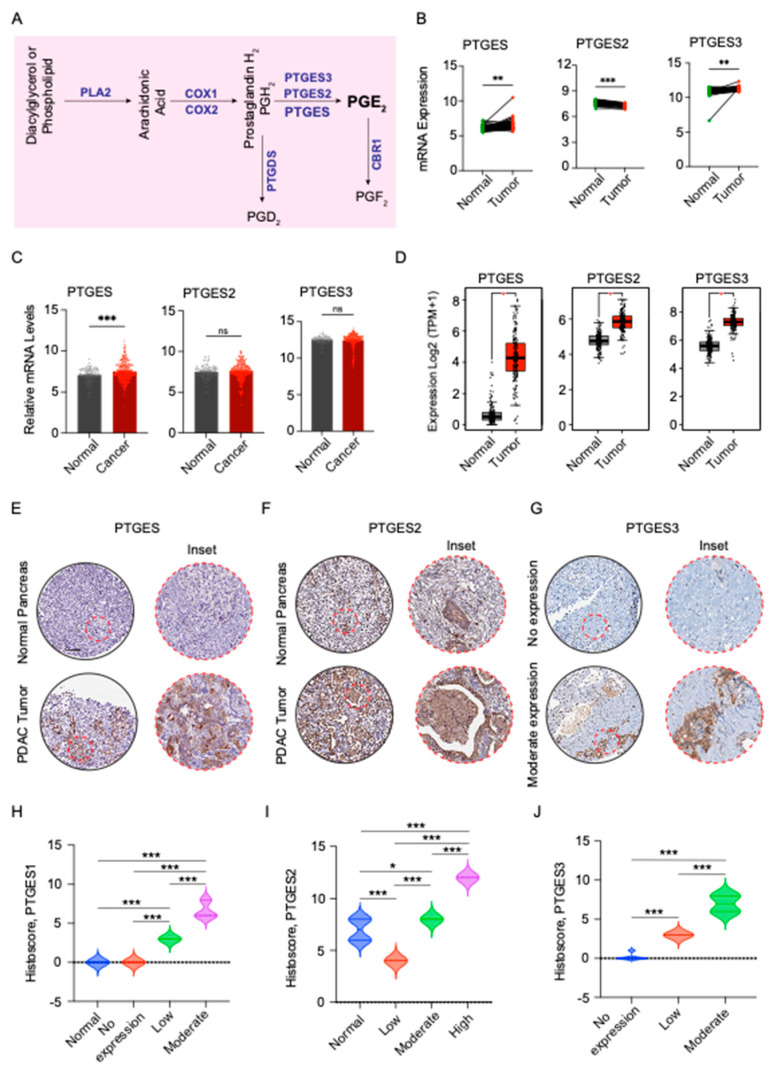
Prostaglandin E synthase expression in pancreatic cancer. (**A**) Schematic illustration of the prostaglandin metabolic pathway. The metabolites are shown in black and the enzymes in blue. (**B**) The relative mRNA expression of the PTGES, PTGES2, and PTGES3 genes in 39 paired normal pancreas and PDAC tumor tissues from the GDS4103 microarray study. (**C**) The relative mRNA expression of the PTGES, PTGES2, and PTGES3 genes in normal pancreas (n = 105) and PDAC tumor tissues (n = 324) from the GENT U133A microarray study. (**D**) The relative mRNA expression of the PTGES, PTGES2, and PTGES3 genes in normal pancreatic tissues from the GTEx study (n = 171) and PDAC tumor tissues (n = 179) from the TCGA PAAD samples. (**E**–**G**) Representative immunohistochemical images of PTGES (**E**), PTGES2 (**F**), and PTGES3 (**G**) proteins in tissue sections of normal and pancreatic tumor tissues with inset of zoomed images. The scalebar is 200 µm. (**H**–**J**) The composite histoscores of PTGES (**H**), PTGES2 (**I**), and PTGES3 (**J**) protein expression in normal pancreas tissue and PDAC tumor tissues from the Protein Atlas Database. The *p*-values are denoted by * < 0.05, ** < 0.01, and *** < 0.001, ns not significant.

PTGES is required for the synthesis of PGE2 metabolites, which support epithelial tumor aggressiveness. PG metabolites have been shown to be elevated in multiple cancers, including pancreatic cancer [23]. PTGES is a critical enzyme that catalyzes the conversion of prostaglandin H2 into a prostaglandin E2 metabolite that is exported out of the cell (Figure 1A). In this study, we assessed the expression of the three PTGES isoforms in PDAC patient samples utilizing publicly available datasets. All three isoforms were found to be expressed more in PDAC cancer tissue samples than in the matched normal tissues from the GDS4013 study (Figure 1B). However, another study comprising a larger patient cohort showed only the PTGES isoform to be significantly increased in pancreatic cancer tissues compared to normal pancreatic tissues (Figure 1C). Further, the mRNA expression of all the three PTGES isoforms was significantly increased in pancreatic cancer tissues from The Cancer Genome Atlas (TCGA) study, compared to the normal pancreatic tissues from the GTEx study. Notably, the maximal difference in expression between cancerous and normal tissue was observed for the PTGES gene (Figure 1D). To further validate our transcription-based changes, we evaluated the expression of PTGES proteins in pancreatic tumors and normal pancreatic tissue sections from the protein atlas database. PTGES protein expression was found to be significantly higher in pancreatic tumor sections compared to normal pancreatic tissue sections (Figure 1E,H). A similar expression pattern was noted for the PTGES2 protein in pancreatic tumors (Figure 1F,I). We noted higher PTGES protein expression in multiple pancreatic tumor sections. However, we could not compare the expression with normal pancreatic tissue due to the non-availability of normal pancreatic tumor sections in the Protein Atlas Database (Figure 1G,J). These data from multiple databases suggest higher expression of PTGES proteins in pancreatic tumors, and specifically, PTGES protein may have oncogenic functions.

### 2.2. PTGES Expression Is Associated with Poor Survival of PDAC Patients

**Figure 2:**
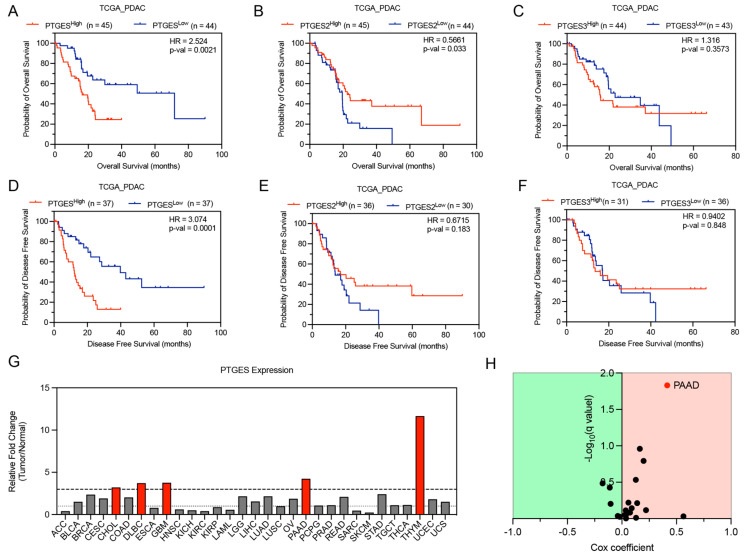
Prostaglandin E synthase-based prediction of pancreatic cancer patient survival. (**A**–**C**) Kaplan–Meier survival plots showing overall survival of pancreatic cancer patients based on expression of PTGES (**A**), PTGES2 (**B**), and PTGES3 (**C**) mRNA in upper and lower quartiles from the TCGA PAAD patient data. (**D**–**F**) Kaplan–Meier survival plots showing disease-free survival of pancreatic cancer patients based on quartile expression of PTGES (**D**), PTGES2 (**E**), and PTGES3 (**F**) mRNA from the TCGA PAAD patient data. (**G**) The relative fold change in expression of the PTGES gene in tumor tissues compared to normal tissues in multiple cancers from the TCGA database. The dashed line represents a fold change value of 3. The cancer type with fold change > 3 is highlighted in red. (**H**) Volcano plot showing the significant oncogenic function of PTGES in PAAD cancer (red dot) compared to other cancers from the TCGA database. The Cox coefficient of PTGES survival is plotted with the q-value significance.

Since PTGES expression was higher in pancreatic cancer tissues, we next evaluated whether any of the PTGES isoforms could predict the prognosis of pancreatic cancer patients. We performed quartile stratification of pancreatic cancer patients based on PTGES expression in a PAAD cohort of TCGA patients. Higher expression of the PTGES gene was significantly associated with worse prognosis in the overall survival of PDAC patients, whereas PTGES2 expression was significantly associated with better prognosis of PDAC patients (Figure 2A,B). PTGES3 gene expression was not correlated with overall survival of PDAC patients (Figure 2C). We further checked whether the PTGES genes were correlated with disease-free survival of pancreatic cancer patients. As expected, PTGES gene expression was significantly associated with worse prognosis in disease-free survival of PDAC patients, whereas PTGES2 expression showed a trend toward better prognosis, which was not significant (Figure 2D,E).

The PTGES3 gene was not associated with disease-free survival of PDAC patients (Figure 2F). Based on tissue expression and survival analysis, only the PTGES gene was significantly increased in pancreatic cancer patients and associated with worse prognosis of patients. We further tested whether PTGES could act as an oncogene in other types of cancers. Comparison of PTGES expression between normal tissues and cancer patient tissues of multiple cancers revealed significantly higher expression (>3-fold change) of PTGES mRNA in cholangiocarcinoma, diffuse large B cell lymphoma, glioblastoma, pancreatic cancer, and thymoma (Figure 2G). We then compared the expression of the PTGES gene with overall survival in multiple cancers. Although the PTGES gene was shown to be oncogenic in multiple cancers, only pancreatic cancer had a significant q-value Cox coefficient (Figure 2H). In summary, our data indicated that PTGES has an oncogenic function and is associated with poor prognosis in pancreatic cancer patients.

### 2.3. RNA-Seq Identifies Association between PTGES Expression and Metabolic and Immune Pathways

**Figure 3:**
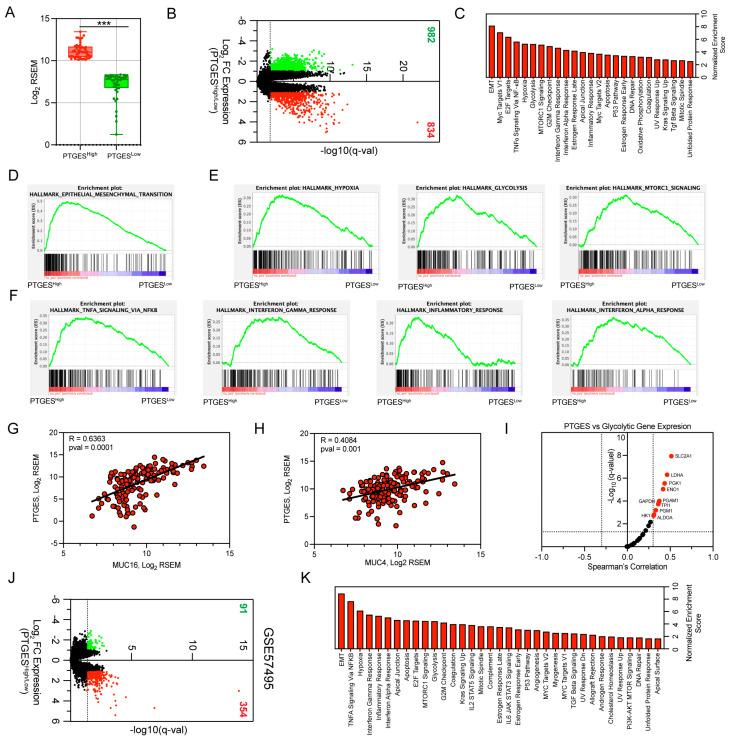
RNA-Seq analysis of pancreatic ductal adenocarcinoma patients based on PTGES expression. (**A**) Boxplot showing the expression of PTGES in PTGES^High^ (n = 45) and PTGES^Low^ (n = 44) pancreatic cancer patients from the TCGA database. (**B**) Volcano plot of differentially expressed genes between PTGES^High^ and PTGES^Low^ patient samples. A total of 834 significantly upregulated genes are shown in red, while 982 significantly downregulated genes (fold change cut-off > 2 and q-value < 0.05) are shown in green. (**C**) Barplot of significantly enriched hallmark pathways based on GSEA analysis between the PTGES^High^ group and the PTGES^Low^ group. (**D**) GSEA plot of the epithelial-mesenchymal transition pathway. (**E**) GSEA plots of metabolic pathways (hypoxia, glycolysis, and MTORC1 signaling) enriched in PTGES^High^ patient data. (**F**) GSEA plots of immune pathways positively correlated with PTGES expression in pancreatic cancer patients. (**G**,**H**) Spearman’s rank correlation plots of MUC16 (**G**) and MUC4 (**H**) oncoproteins with PTGES expression in pancreatic cancer patients. (**I**) Volcano plot of Spearman’s rank correlation of glycolytic enzyme expression with PTGES expression in pancreatic cancer samples from the TCGA PAAD database. (**J**) Volcano plot of differentially expressed genes between PTGES^High^ (n = 15) and PTGES^Low^ (n = 15) pancreatic cancer patient samples from GSE57495. A total of 354 significantly upregulated genes are shown in red, while 91 significantly downregulated genes (fold change cut-off > 2 and q-value < 0.05) are shown in green. (**K**) Barplot of positively enriched hallmark pathways based on GSEA analysis between the PTGES^High^ group and the PTGES^Low^ group from GSE57495. The q-value significant (<0.05) pathways are shown in red.

To explore the role of PTGES in mediating oncogenic signaling in pancreatic cancer, we analyzed differentially expressed genes between PTGES^High^ and PTGES^Low^ patient groups. The stratification of pancreatic cancer patients for RNA-Seq analysis was performed using quartile-based overall survival, and PTGES expression was significantly different between the groups (Figure 3A). RNA-Seq analysis identified 834 significantly upregulated genes and 982 downregulated genes with a two-fold change in cut-off value and q-value significance of <0.05 in the PTGES^High^ group compared to the PTGES^Low^ group (Figure 3B). To identify the PTGES-altered pathways in PDAC, we performed Gene Set Enrichment Analysis (GSEA) between the two groups. While many pathways were positively enriched with high PTGES expression, only a few pathways were negatively enriched (Figure 3C).

Since we predicted that PTGES could be an oncogene, we therefore focused on positively enriched pathways. Our analysis identified epithelial-mesenchymal transition (EMT) as the top enriched pathway, suggesting a role of PTGES in metastasis (Figure 3D). Interestingly, we observed a variety of metabolic pathways, including hypoxia, glycolysis, and MTORC1 signaling, that were positively enriched in the PTGES^High^ group (Figure 3E). Both hypoxia and glycolysis are well known to support proliferation of cancer cells and induce oncogenic survival [24,25]. Further, we also identified a variety of immune response pathways, including TNFα signaling via NF-κB, interferon gamma response, inflammatory response, interferon alpha response, and TGF beta signaling, to be significantly enriched in the PTGES^High^ group (Figure 3F). Moreover, mucin (MUC) proteins were among the top upregulated proteins in the analysis. Mucin proteins are a group of heavily glycosylated proteins that are oncogenic in nature [26]. Studies have shown the critical role of mucins in pancreatic cancer pathogenesis [27]. MUC16, MUC4, and MUC21 proteins showed significant, positive correlations with PTGES expression (Figure 3G,H and Figure S1A). We further correlated the expression of glycolytic pathway enzymes with PTGES expression and found many of the glycolytic genes to be positively correlated. SLC2A1, LDHA, PGK1, ENO1, PGAM1, GAPDH, TPII, PGM1, HK1, and ALDOA were significantly correlated with PTGES expression (Figure 3I and Figure S1B–L). Further, we validated the gene expression regulation by PTGES in another pancreatic adenocarcinoma patient cohort from the GSE57495 study (Figure 3J). Consistently, similar oncogenic pathways were found to be positively associated with higher PTGES expression (Figure 3K and Figure S1M–O). However, very few pathways were negatively enriched in PDAC patients with higher PTGES expression. Notably, pancreatic beta cells and oxidative phosphorylation pathways were significantly regulated (Figure S1P,Q). Overall, our data here propose that PTGES could be a key regulator of metabolic and immune pathways in pancreatic cancer.

### 2.4. PTGES Expression Is Associated with a Higher Mutation Burden in Key Driver Genes

**Figure 4:**
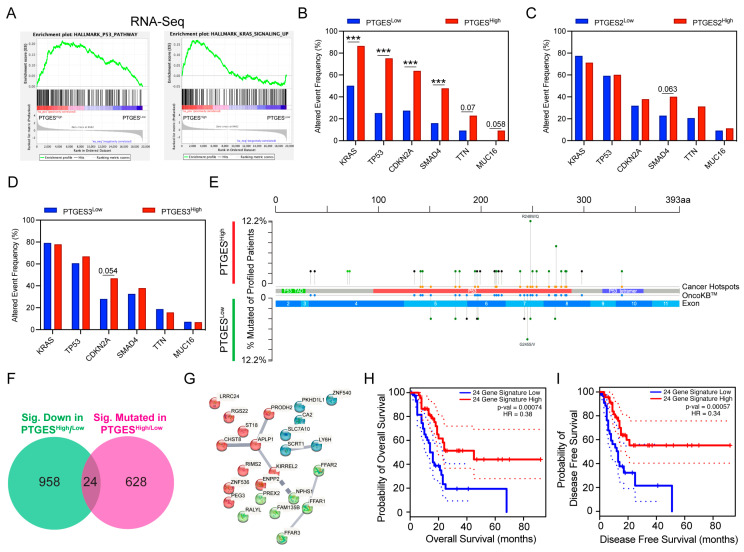
The mutational landscape of pancreatic cancer patients based on PTGES expression. (**A**) GSEA plots from the RNA-seq data based on GSEA expression showing positive enrichment of the P53 pathway and KRAS signaling pathway. (**B**–**D**) The mutational profiles of key driver genes (KRAS, TP53, CDKN2A, SMAD4, TTN, and MUC16) in pancreatic cancer between the patients with high and low expression of PTGES (**B**), PTGES2 (**C**), and PTGES3 (**D**) in pancreatic cancer patients from the TCGA cohort. (**E**) The mutational landscape of the P53 gene between the PTGES^High^ and PTGES^Low^ patient groups showing the location and higher incidences of mutations in the PTGES^High^ group. Additional tracks show the cancer hotspot mutations. (**F**) Venn diagram shows 24 genes, the expression of which is impacted by the mutations. (**G**) The String gene network showing the subset of genes impacted by mutations. (**H**) The prognostic impact of the 24-gene signature on overall survival of pancreatic cancer patients stratified by quartile expression. (**I**) The prognostic impact of the 24-gene signature on disease-free survival of pancreatic cancer patients stratified by quartile expression. The *p*-values are denoted by *** < 0.001.

The P53 pathway and KRAS signaling pathways were two other pathways positively correlated with higher PTGES expression (Figure 4A). Mutations in KRAS and P53 are two common drivers of pancreatic cancer pathogenesis and progression [5,6]. Interestingly, when we compared the mutational burden between the two groups, the PTGES^High^ group had a significantly higher mutational load. We postulated that the mutational burden is enriched in the key genes harboring driver mutations. KRAS, TP53, CDKN2A, SMAD4, TTN, and MUC16 are key genes that are frequently mutated in pancreatic cancer patients and are critical for cancer progression [6]. Consistent with our hypothesis, we observed higher mutations in key driver genes in patients with higher PTGES expression compared to patients with low PTGES expression (Figure 4B). Interestingly, the trend of higher mutational burden in key genes was specifically associated with the PTGES gene only and was not correlated with PTGES2 and PTGES3 gene expression (Figure 4C,D). Further analysis revealed that, while a number of genes were significantly enriched between the two groups, only the P53 gene mutational burden was altered based on q-value significance. Many of the P53 mutations found in the PTGES^High^ patient group were cancer hotspot mutations and enriched in missense mutations (Figure 4E). Next, we compared *p*-value significant mutated genes with significantly downregulated genes in RNA-Seq analysis to identify the genes impacted by mutations. To our surprise, the expression of a very small subset of genes was impacted by mutations (Figure 4F). The identified 24-gene signature, predicted to be a group of tumor suppressor genes, did not form a strong, inter-connected interaction network (Figure 4G).

However, quartile-based expression of this 24-gene signature was associated with better prognosis of pancreatic cancer patients. The gene signature was significantly associated with overall and disease-free survival of patients (Figure 4H,I). We further evaluated whether mutational burden in pancreatic cancer patients could predict their prognosis. Although PTGES expression was associated with higher mutational burden, quartile-based stratification of PTGES expression was significantly associated with worse prognosis in PDAC patients (Figure S2). Our data here identify a link between higher mutational burden in key driver genes for pancreatic cancer and PTGES expression.

### 2.5. PTGES Is Correlated with Glycolytic and Immune Pathways in PDAC Patients Potentially via DNA Methylation

**Figure 5:**
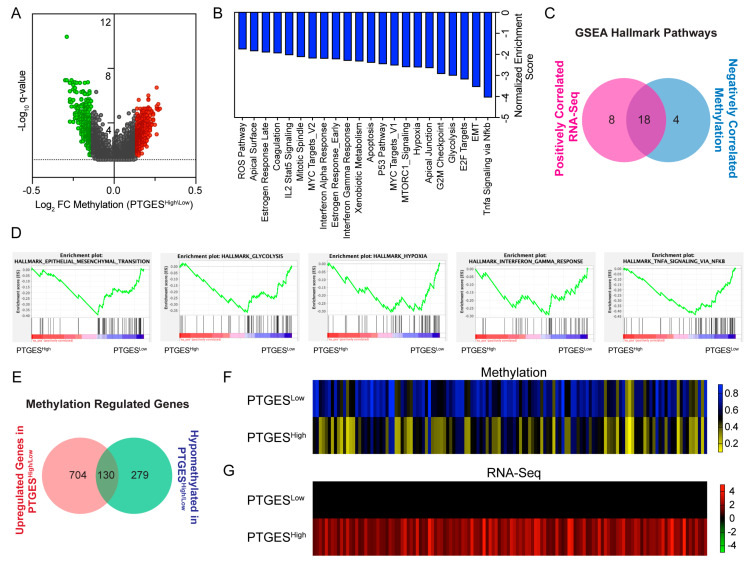
Correlation of DNA methylation with mRNA expression in PDAC patients based on PTGES expression. (**A**) Volcano plot and (**B**) barplot showing GSEA pathway enrichment analysis of significantly hypo- and hypermethylated genes between the PTGES^High^ and PTGES^Low^ groups in pancreatic cancer patients. (**C**) Venn diagram of GSEA hallmark pathways between positively enriched pathways in RNA-Seq data and negatively enriched pathways in methylation data correlated with high PTGES expression. (**D**) GSEA plots of selected common regulated pathways. The plots shown are negatively enriched pathways with PTGES expression from methylation data. (**E**) Venn diagram shows the genes regulated by methylation. The upregulated genes in RNA-Seq data are compared with hypomethylated genes from the methylation analysis. (**F**) Heatmap showing methylation status of the commonly identified 130 genes between the PTGES^High^ and PTGES^Low^ patient groups. The color key shows the percentage methylation change. (**G**) Heatmap showing expression changes of the commonly identified 130 genes between the PTGES^High^ and PTGES^Low^ patient groups. The color key shows log2 fold changes in expression.

Epigenetic reprogramming is a well-known paradigm of pancreatic cancer cells that facilitates pancreatic cancer development and progression. In particular, DNA methylation negatively regulates gene expression and is often observed in the CpG islands located in the promoters of tumor suppressor genes [28,29]. We hereby investigated the role of PTGES in DNA-methylation-regulated gene expression. A total of 402 genes were significantly hypermethylated, and 409 genes were significantly hypomethylated in patients with higher expression of PTGES (Figure 5A). Pathway enrichment analysis of DNA-methylation-regulated genes revealed a total of 22 pathways to be negatively enriched with higher PTGES expression (Figure 5B). Since we focused on oncogenic pathways, we were interested in analyzing negatively enriched hypomethylated pathways. To find methylation-regulated oncopathways, we compared significantly enriched positive pathways from RNA-seq data with negatively enriched methylated pathways. Interestingly, the majority of [18] pathways were regulated by methylation (Figure 5C). The EMT pathway, metabolic pathways, immune-related pathways, and cell cycle pathways were among the commonly regulated pathways. Metabolic pathways, such as hypoxia and glycolysis, and immune-related pathways, such as TNFα signaling via NF-κB, interferon-gamma response, interferon alpha response, and IL2-STAT5, were among the major hypomethylated pathways (Figure 5B,D). Further, we identified methylation-upregulated genes by comparing significantly upregulated genes (fold change > 2) from RNA-Seq data with significantly hypomethylated genes. A total of 11.7% (130) of genes were regulated by DNA methylation (Figure 5E). Among the regulated genes, genes belonging to glycolysis, immune pathways, and MUC4 were notable. However, we could not find genes belonging to the DNA methylation pathway in this analysis (Figure 5F,G). Our data here reveal a possible epigenetic mechanism for PTGES-regulated oncopathways.

### 2.6. PTGES Expression Is Correlated with Immune Infiltration in PDAC Patients

**Figure 6:**
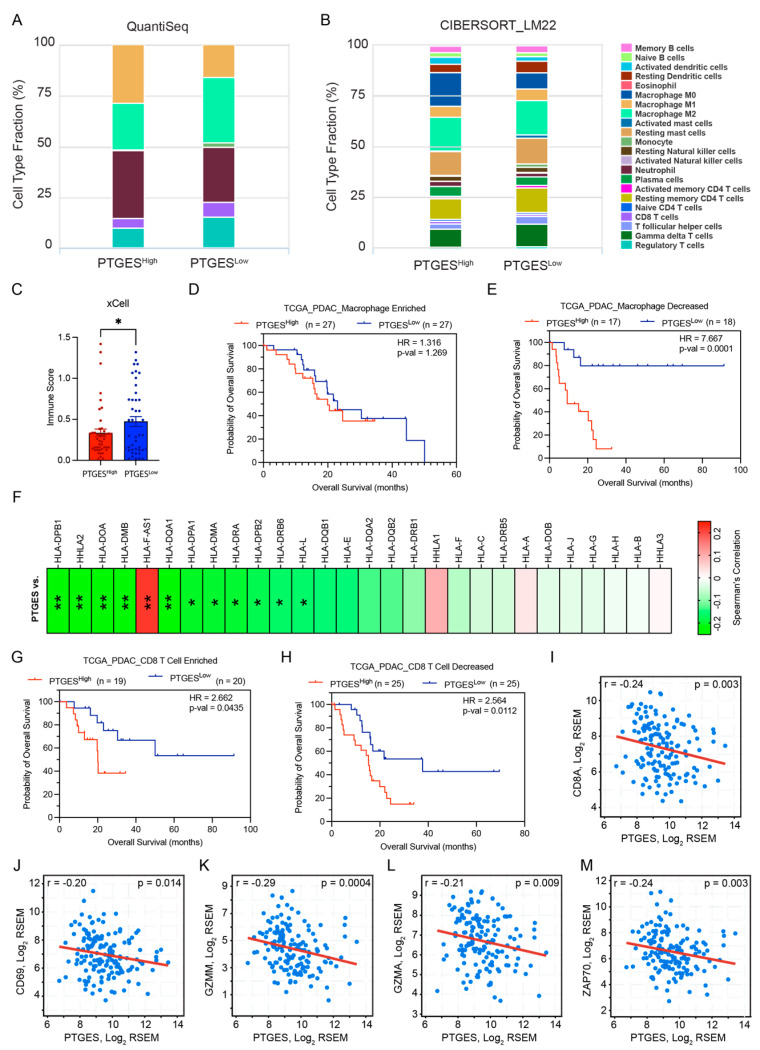
PTGES is associated with an altered immune microenvironment in pancreatic cancer. (**A**) The percentage distribution of six immune cells between the PTGES^High^ and PTGES^Low^ patient groups based on QuantiSeq analysis from the Cancer Immunome Database. (**B**) The percentage distribution of multiple immune cells between the PTGES^High^ and PTGES^Low^ patient groups based on CIBERSORT analysis using the LM22 signature from the Cancer Immunome Database. (**C**) The total immune score calculated through xCell software between PTGES^High^ and PTGES^Low^ PDAC patients. (**D**,**E**) Kaplan–Meier survival plots of overall survival differences between upper and lower quartiles of PTGES expression in PDAC patients with enriched (**D**) or decreased (**E**) macrophage populations. (**F**) Heatmap showing Spearman’s rank correlation of MHC I and II gene expression with PTGES expression in pancreatic cancer patients from the TCGA PAAD data. The *p*-value significance of correlations is shown inside the cells. (**G**,**H**) Kaplan–Meier survival plots of overall survival differences between upper and lower quartiles of PTGES expression in PDAC patients with enriched (**G**) or decreased (**H**) CD8 T cell populations. (**I**–**M**) Spearman’s rank correlation plots of the CD8A gene and other marker genes (CD69, GZMM, GZMA, and ZAP70) activated in CD8+ T cells with PTGES expression in PDAC patients. The *p*-values are denoted by * < 0.05, and ** < 0.01.

The immune microenvironment is critical for supporting oncogenesis, and the interaction of cancer cells with immune cells is pivotal for tumor progression [30]. Since immune-regulated pathways were prominent in our RNA-Seq and methylation analyses, we investigated whether PTGES could reprogram the immune microenvironment in pancreatic cancer. We determined an immune subtype population from bulk RNA-sequencing data of pancreatic tumors with high and low expression of PTGES in the PAAD cohort from TCGA using QuantiSeq software and Cibersort analysis using LM22 signature. Multiple immune cells were altered between the groups. We observed a substantial decrease in the M1 macrophage population, whereas an increase in the M2 macrophage population was observed in the PTGES^Low^ group. The macrophage population shift was contrary to the known function of tumor burden regulation. However, an increase in the CD8^+^ T cell population was correlated with lower expression of PTGES, indicating a cytotoxic function of CD8^+^ T cells in reducing tumor burden (Figure 6A,B). The immune score prediction using xCell software suggested a significant association between increased immune cell infiltration in tumors and low PTGES expression (Figure 6C).

Further, we investigated how PTGES expression influences the prognostic outcomes in overall survival of pancreatic cancer patients in multiple immune cells with enriched or depleted microenvironments. Higher expression of PTGES was a better predictor of worse prognosis of PDAC patients in multiple immune cell-depleted samples compared to macrophages, B cells, and CD4+ T cell-enriched samples (Figure 6D,E and Figure S3A–D). In contrast to these immune subtypes, PTGES expression predicted worse prognosis of PDAC patients in eosinophil-enriched samples, whereas no apparent trend was seen in eosinophil-diminished samples (Figure S3E,F). To further understand the significance of increased M1 macrophage populations in PTGES-high tumors, we checked the correlation of PTGES with major histocompatibility complex (MHC) I and II pathway genes. Linear regression analysis showed a negative correlation in most of the HLA genes in pancreatic cancer samples (Figure 6F). This outcome suggests that PTGES could downregulate the MHC pathway to decrease antigen presentation and may aid tumor progression. We further assessed the role of increased CD8+ T cells in PTGES-low tumors and their role in the prognosis of PDAC patients. Similar to previous findings, higher PTGES expression predicted a worse prognosis of PDAC patients with decreased CD8+ T cell infiltration compared to samples with enriched CD8^+^ T cells (Figure 6G,H). Furthermore, a CD8^+^ T cell marker, CD8A, was negatively correlated with higher PTGES expression, revealing an increase in CD8^+^ T cells in PTGES-low samples (Figure 6I). The CD8+ T cell activation markers CD69, GZMM, GZMA, GZMK, GZMH, and ZAP70 also showed a significant, negative correlation with PTGES expression (Figure 6J–M and Figure S3G,H). In conclusion, our data suggest that the smaller tumor burden in PTGES-low tumor samples is associated with higher infiltration and activation of cytotoxic CD8^+^ T cells.

## 3. Discussion

The prostaglandin metabolic pathway is critical for inflammation, tissue homeostasis, angiogenesis, migration, invasion, and tumorigenesis. Dysregulated prostaglandin metabolism has been reported in multiple cancers [22,31]. Prostaglandin pathway metabolites, especially PGE2, were elevated in tumor tissues from mouse models and pancreatic cancer patient samples [19]. The cyclooxygenase enzyme is a well-studied enzyme in cancer that converts arachidonic acid into prostaglandin H2, which is further converted into five primary prostanoids [32]. Clinical trials using NSAID inhibitors for the COX enzyme have yielded limited benefits due to higher risks of myocardial infarction and gastrointestinal toxicity [33,34]. For this reason, the focus has shifted to identifying new targets in the prostaglandin metabolic pathway. Prostaglandin E2, a product of PTGES, is secreted by cancer cells and binds to G protein-coupled receptors on cancer cells and other cells of the TME [35]. Among all cancer types, PTGES is prominently associated with worse prognosis in pancreatic cancer. We therefore emphasized finding the significance of the PTGES gene in pancreatic cancer. PTGES expression is specifically increased in pancreatic tumor tissues compared to normal pancreatic tissue. In a recent study, PTGES was identified as one of six metabolism-related gene-based prognostic signatures in PDAC patients correlated with worse outcomes. The prognostic signature-based differential expression analysis identified alterations in the metabolic pathways critical for tumor proliferation and survival [36].

Alterations in metabolic pathways support cancer cell growth in nutrient-limiting conditions such as hypoxia [17]. The Warburg effect is a well-established mechanism supporting the bioenergetic demands of a proliferating cancer cell [37]. Interestingly, our analysis identifies associations between PTGES and glycolytic metabolism and hypoxia signaling. Cancer cells activate HIF-1α signaling to manage hypoxic stress in the TME and directly regulate glycolysis [38,39]. Previously, PTGES was shown to be an HIF-inducible gene in esophageal cancer. Moreover, PGE2 production was enhanced in an HIF-1α-dependent manner under hypoxia [40]. HIF-2α signaling was also implicated in activation of the prostaglandin pathway in colon carcinoma [41]. Based on these data, we speculated on the possibility of a positive feedback loop between PTGES and HIF signaling in a hypoxic microenvironment. In addition to hypoxia-mediated regulation of PTGES isoforms, miR-155-mediated upregulation of PTGES has also been reported in triple-negative breast cancer [42]. Apart from regulating metabolism, hypoxia also regulates migration, invasion, and metastasis [43]. Accordingly, we observed that EMT was one of the top regulated pathways by PTGES in our analysis. Recently, PTGES was identified as a candidate gene in a proteogenomic screen for pancreatic cancer. Knock-down of PTGES in AsPC1 and PANC1 PDAC cells was shown to regulate spheroid growth, migration, and invasion in vitro [44]. In conclusion, we propose that PTGES may support oncogenic transformation and metastasis by modulating cancer cell metabolism and hypoxia signaling.

Pancreatic cancer tumorigenesis is linked with frequent genetic mutations in KRAS, TP53, CDKN2A, and SMAD4. Activating mutations in the KRAS oncogene were observed in 92–95% of patients and constitute major steps in tumor initiation and progression [45]. Additional mutations in the tumor suppressor genes TP53, CDKN2A, and SMAD4 further aggravate the progression of tumors. The genetic alterations in these key driver genes mediate oncogenic functions by rewiring cellular metabolism [6]. We therefore investigated whether there is any link between PTGES and mutations in common driver genes found in pancreatic cancer patients. Pancreatic cancer patients with high PTGES expression correlated with a high mutational burden. This mutational enrichment was specifically observed with the PTGES isoform but not with PTGES2 and PTGES3 expression. Interestingly, TP53 was the most significantly mutated gene, with a very high number of cancer hotspot mutations. TP53 mutations facilitate malignant transformation into tumors from PanIN lesions, and they are implicated in lymph node metastasis [4]. Although it is not clear how PTGES induces mutations in key driver genes, it could be a potential mechanism of oncogenesis and requires further investigation in the future. Further, a comparison of differentially regulated genes with mutated genes did not yield a greater overlap, suggesting that widespread mutational changes do not translate into expression-level changes.

Previous studies have demonstrated that metabolic reprogramming in pancreatic cancer cells is intricately associated with epigenetic modifications [46,47]. Aberrant genome-wide DNA methylation is one such epigenetic mechanism that regulates transcriptional changes associated with tumorigenesis and metastasis [48]. Aberrant DNA methylation has also been proposed as a prognostic marker for pancreatic cancer. Alterations in the promoter methylation level of genes affect the transcription of oncogenes and tumor suppressors, thus regulating tumorigenesis [49]. We therefore checked whether PTGES-regulated transcriptional changes are regulated by DNA methylation. Our focused comparative analysis of positively regulated genes by PTGES with hypomethylated genes identified a greater overlap, suggesting that decreased promoter methylation could be a possible mechanism for increased transcription of genes. More than 80% of pathways were commonly regulated, including EMT and metabolic pathways such as hypoxia and glycolysis, which are well-known factors promoting oncogenesis. A variety of immune pathways, including inflammatory response and interferon pathways, were also regulated in a methylation-dependent manner. A previous study in colorectal cancer identified DNA methylation as regulating multiple genes in the prostaglandin pathway [50]. However, our study revealed the presence of methylated-differentially expressed genes that could be critical for oncogenic transformation in pancreatic cancer.

Prostanoids have been known to induce inflammation and inhibit anti-tumor immunity. However, their immunostimulatory function in dendritic cells, macrophages, and T_h_ cells was recently recognized [35]. PGE2 suppresses the effector function of innate and adaptive immune cells, including CD8+ T cells, NK cells, macrophages, dendritic cells, and T helper cells [51]. In a lung metastatic mice model, PGE2 signaling activation recruited immunosuppressive MDSC cells to the tumor and led to dysfunction of cytotoxic CD8^+^ T cells [52]. In another study utilizing single-cell sequencing data of pancreatic cancer, PTGES3 expression was prominently seen in multiple immune cells, whereas PTGES expression was mostly confined to fibroblast and tumor cells [19]. In our analysis, PTGES was found to be associated with many important genes belonging to immune pathways implied in cancer and immune cell crosstalk. Linear regression analysis showed that PTGES downregulated antigen presentation machinery, which may limit cancer antigen presentation to immune cells. PTGES expression also correlated negatively with immune cell dysfunction and especially inhibited cytotoxic CD8+ T cells. Importantly, PTGES expression associates better with poor prognosis of pancreatic cancers and patient survival in an immune-deficient microenvironment. Therefore, we postulate that PTGES could limit the infiltration of immune cells into the TME and suppress anti-tumor immunity.

To summarize, our comprehensive study identified that PTGES could be a major regulator of oncogenic signaling and a prognostic marker for pancreatic cancer patients. PTGES expression is positively correlated with the glycolytic phenotype of cancer cells and can potentially fuel their proliferation and metastasis. PTGES may further support oncogenesis by suppressing anti-tumor immunity. Thus, our study here provided valuable insights into the plausible mechanism of PTGES-mediated oncogenic effects in pancreatic cancer. While our computational analysis identified multiple potential modes of oncogenic regulation by PTGES, future studies utilizing experimental approaches are required to validate our findings.

## 4. Materials and Methods

### 4.1. Data Source and Collection

Normalized RNA sequencing (fragments per kilobase million, FPKM) for TCGA PAAD were obtained from TCGA (https://portal.gdc.cancer.gov/, accessed on 12 January 2023). The raw data, processed data, and clinical data can be found in the legacy archive of the National Cancer Institute’s Genomic Data Commons (GDC) (https://portal.gdc.cancer.gov/legacy-archive/search/f accessed on 12 January 2023). The clinical information of the TCGA PAAD patients was obtained from the Memorial Sloan Kettering Cancer Center cBioPortal (http://www.cbioportal.org accessed on 12 January 2023) [53,54]. A total of 179 PAAD tissues from the TCGA database were analyzed. The top and bottom quartiles of PDAC patients based on PTGES expression were selected and classified as PTGES^High^ and PTGES^Low^ groups for further analysis. PTGES, PTGES2, and PTGES3 mRNA expression data from human pancreatic cancer patients were downloaded from the GENT2 database (http://gent2.appex.kr/gent2/ accessed on 29 January 2023) [55]. The messenger RNA (mRNA) expression of all the PTGES isoforms in matched human normal and PDAC tissues was obtained from the GEO database (GDS4103). The comparative expression of the PTGES isoforms between normal pancreas tissues from the GTEx portal and tissue from the pancreatic ductal adenocarcinoma patients from the TCGA database were compared using GEPIA 2 software and expressed as log2 (TPM+1) values [56].

### 4.2. Immunohistochemical Analysis of PTGES Isoforms from Protein Atlas

The immunohistochemical images showing the protein expression levels of PTGES, PTGES2, and PTGES3 were obtained from Human Protein Atlas Database for normal and pancreatic cancerous tissues (https://www.proteinatlas.org accessed on 7 February 2023). IHC staining scores for PTGES, PTGES2, and PTGES3 were calculated by multiplying scores for the intensity of staining and the extent of staining (percentage of stained cells) [57]. The parameter used to score the tissue staining based on intensity was as follows: 0-no staining, 1-weak, 2-moderate, and 3-high staining. The criteria used to score the staining of the tissues based on the percentage of cells stained were as follows: 0—0% stained cells; 1—1–25% stained cells; 2—26–50%; 3—51–75%; and 4—>75%.

### 4.3. Survival Analysis of PTGES Isoforms in TCGA PAAD Cohort

To determine the independent prognostic value for PTGES isoforms, we performed Kaplan–Meier survival analysis in the TCGA PAAD patient cohort by the Mantel–Cox log-rank test to determine the significance of overall survival and disease-free survival [58]. The samples were divided into high-expression or low-expression groups according to the top and bottom quartiles of gene expression, respectively.

### 4.4. RNA-Seq Analysis of PTGES^High^ and PTGES^Low^ Patient Groups in Pancreatic Cancer

The log2 ratio of the mean mRNA expression values (RSEM) of the genes between the PTGES^High^ and PTGES^Low^ groups in the TCGA PAAD samples were obtained from the cBioPortal (http://www.cbioportal.org accessed on 23 January 2023) [53,54]. The significance of the expression ratio was calculated using the Benjamini–Hochberg procedure. The genes with log2 fold-change values greater than 1 were considered upregulated, whereas expression values less than −1 were considered downregulated genes. Genes with q-values < 0.05 were considered significant. The linear regression analysis of PTGES mRNA expression with MUC oncoproteins or glycolytic gene expression in TCGA PAAD samples was computed using Spearman’s rank correlation analysis.

### 4.5. RNA-Seq Data Validation in PDAC Patient Cohort from GSE57495 Study

We obtained the gene expression data of 63 early-stage pancreatic ductal adenocarcinoma tumor samples using the microarray data based on GPL15048 (Rosetta/Merck Human RSTA Custom Affymetrix 2.0 microarray HuRSTA 2a520709.CDF) (Affymetrix, Tampa, FL, United States). The data were accessed at Gene Expression Omnibus (GEO; http://www.ncbi.nlm.nih.gov/geo/ accessed on 12 March 2023), from the GSE57495 study. The PDAC patient cohort was stratified into PTGES^High^ and PTGES^Low^ groups based on the PTGES gene expression sorted by quartile. The differential gene expression analysis between the two groups was performed by GEO2R using the limma method. The statistical significance was calculated by the Benjamini–Hochberg test. The gene set enrichment analysis between the two groups was performed using the hallmark pathways database.

### 4.6. Gene Set Enrichment Analysis of Differentially Expressed Gene

Gene set enrichment analysis of differentially expressed genes was performed between PTGES^High^ and PTGES^Low^ tumor samples. The log-ranked file (.rnk) generated for differentially expressed genes was processed using GSEA, version 4.2.3, with 1000 permutations in the classic scoring scheme using the h.all.v2022.Hs.symbols.gmt database from the Broad Institute. The positively enriched pathways in PTGES^High^ patients were plotted using normalized enrichment scores (NES) and FDR-corrected *p*-values. The differentially expressed genes were depicted by volcano plot using log2 fold change values of expression and -log10 *p*-values of significance in GraphPad Prism Software (version 9.4.1).

### 4.7. Tumor Mutational Burden Analysis in PTGES^High^ and PTGES^Low^ Cohorts

The percentage alteration in somatic mutations in pancreatic tumor samples belonging to the PTGES^High^ and PTGES^Low^ groups was computed using the cBioPortal (http://www.cbioportal.org, accessed on 23 January 2023) [53,54]. The percentage alteration in driver genes of pancreatic cancer between the groups was analyzed based on PTGES isoform expression, and *p*-values were calculated using Fisher’s one-sided exact test. The comparative analysis of significantly mutated genes with *p*-values < 0.05 and significantly downregulated genes from RNA-seq analysis (>2 fold change in expression and q-val < 0.05) identified a common set of 24 genes. The protein interaction network of these 24 genes was constructed using strings software (https://string-db.org accessed on 5 February 2023) with an interaction cut-off score of 0.4. The prognostic value of this 24-gene signature with overall and disease-free survival was computed using GEPIA 2 software [56]. Kaplan–Meier analysis of pancreatic cancer patients’ overall survival with the degree of mutational burden was performed to investigate the prognostic impact of PTGES using the Kaplan–Meier Plotter software (https://www.kmplot.com accessed on 24 January 2023).

### 4.8. DNA Methylation Analysis

The log2 ratio of mean methylation value of the genes between PTGES^High^ and PTGES^Low^ patients from the TCGA PAAD group was computed using the cBioPortal (http://www.cbioportal.org accessed on 23 January 2023) [53,54] The significance of the comparison between the two groups was defined by q-values calculated using the Benjamini–Hochberg procedure. A cut-off value of 10% methylation change with q-value significance < 0.05 was considered to identify hypomethylated and hypermethylated genes. To identify methylation-regulated differential genes, the hypomethylated, q-value significant genes were compared with significantly upregulated differential genes (>2 fold change in expression and q-val < 0.05) from the RNA-Seq data using Venny software (https://bioinfogp.cnb.csic.es accessed on 3 February 2023). The heatmap of percentage methylation changes in the selected genes was plotted in GraphPad Prism Software (version 9.4.1).

### 4.9. Tumor-Infiltrating Immune Cell Prediction in Bulk RNA-Seq Samples

The immune cell composition and density prediction in pancreatic cancer samples belonging to the PTGES^High^ and PTGES^Low^ groups were obtained from the Cancer Immunome Atlas (https://www.tcia.at accessed on 16 February 2023) [59,60]. The composition and fraction of all immune cell types relative to all cells in the pancreatic tumor samples were quantified using QuanTIseq [61] and the CIBERSORT method using the LM22 signature (https://cibersortx.stanford.edu/ accessed on 16 February 2023). The overall immune score prediction in the PTGES^High^ and PTGES^Low^ cohorts was computed using xCell [62]. The linear regression analysis of PTGES gene expression with major histocompatibility complex genes or CD8 T cell activation marker gene expression in the TCGA PAAD cohort was computed using Spearman’s rank correlation in GraphPad Prism software (version 9.4.1).

### 4.10. Survival Analysis of PTGES Gene with Tumor-Infiltrating Immune Cells

The overall survival of pancreatic cancer patients stratified by quartile-based expression of the PTGES gene in immune cell subtype-enriched or -decreased samples was performed using Kaplan–Meier Plotter (https://www.kmplot.com accessed on 22 January 2023) to determine the overall survival [63]. The significance of overall survival between the PTGES^High^ and PTGES^Low^ groups in the context of macrophage, CD8 T cell, B cell, CD4 T cell, and eosinophil infiltration was computed using the Mantel–Cox log-rank test in GraphPad Prism software (version 9.4.1).

### 4.11. Statistical Analysis

The statistical comparison between two groups was analyzed by Student’s *t*-test and more than two groups by one-way ANOVA with Tukey’s post-hoc test. The statistical significance in survival of Kaplan–Meier plots was analyzed the Mantel–Cox log-rank test. The linear regression analysis between the expression of genes was performed using Spearman’s rank correlation. All statistical comparisons were calculated by GraphPad Prism software, version 9. The *p*-values are denoted by * < 0.05, ** < 0.01, and *** < 0.001.

## Data Availability

The data presented in this study are openly available in the TCGA database. The raw data, processed data, and clinical data can be found in the legacy archive of the National Cancer Institute’s Genomic Data Commons (GDC) (https://portal.gdc.cancer.gov/legacy-archive/search/f, accessed on 12 January 2023).

## Supplementary Materials

The following supporting information can be downloaded at: https://www.mdpi.com/article/10.3390/ijms24087304/s1.
