# Supplementary material for: PTGES Expression Is Associated with Metabolic and Immune Reprogramming in Pancreatic Ductal Adenocarcinoma
Source: Int J Mol Sci. 2023 Apr 15;24(8):7304. doi: 10.3390/ijms24087304 (PMC10138618; doi:10.3390/ijms24087304)
Supplement: Supplementary file 1 [file ijms-24-07304-s001.zip › ijms-2281606-supplementary.pdf]

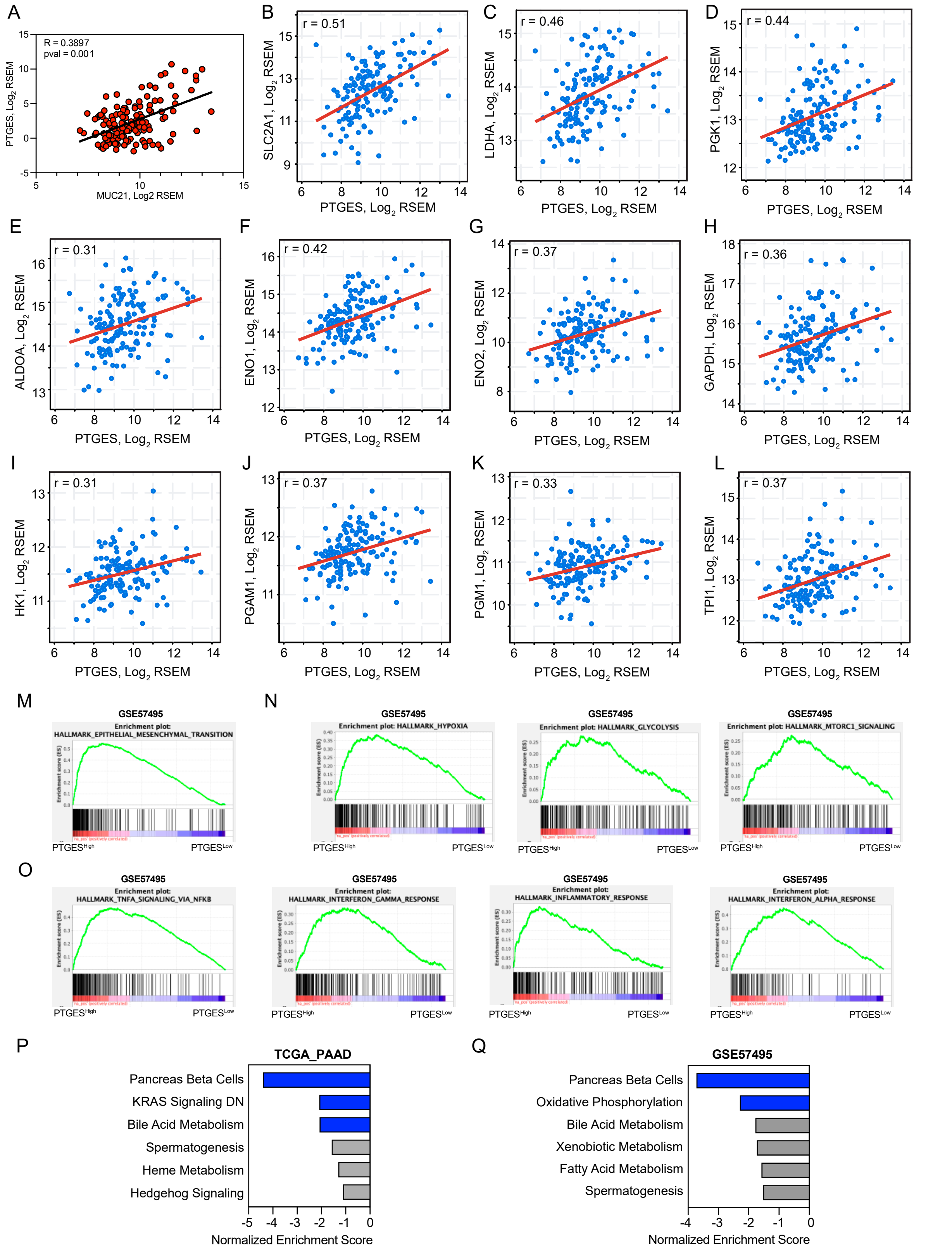

Figure S1. Glycolytic genes and MUC21 are positively correlated with PTGES expression in pancreatic expression. (A) The spearman's rank correlation plot of MUC21 oncoprotein with PTGES expression in PDAC patients. (B-L) The

spearman's rank correlation plots of glycolytic genes (SLC2A1, LDHA, PGK1, ALDOA, ENO1, ENO2, GAPDH, HK1, PGAM1, PGM1, and TPI1) with PTGES expression in PDAC patients from TCGA database. (M) The GSEA plot of epithelial-mesenchymal transition pathway. (N) The GSEA plots of metabolic pathways (hypoxia, glycolysis, and MTORC1 signaling) enriched in PTGESHigh patient data from GSE57495. (O) The GSEA plots of immune pathways positively correlated with PTGES expression in pancreatic cancer patients from GSE57495. (P & Q) The barplot of negatively enriched Hallmark pathways based on GSEA analysis between $PTGES^{High}$ group compared to $PTGES^{Low}$ group from the TCGA_PAAD data (P) and GSE57495 data (Q). The q-value significant (<0.05) pathways are shown in blue.

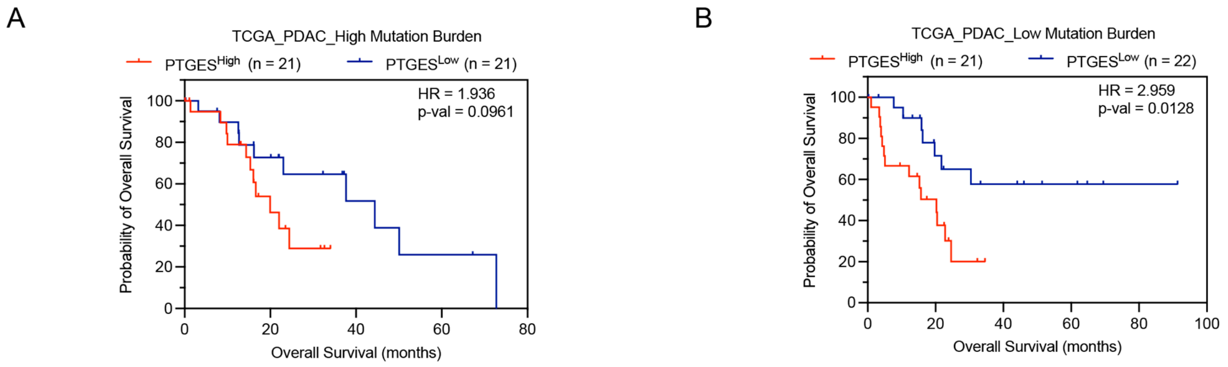

Figure S2. Survival analysis of PTGES with mutational burden in PDAC. (A) The Kaplan-Meier survival plots of overall survival differences between upper and lower quartiles of PTGES expression in PDAC patients with high mutational burden. (B) The Kaplan-Meier survival plots of overall survival differences between upper and lower quartiles of PTGES expression in PDAC patients with low mutational burden.

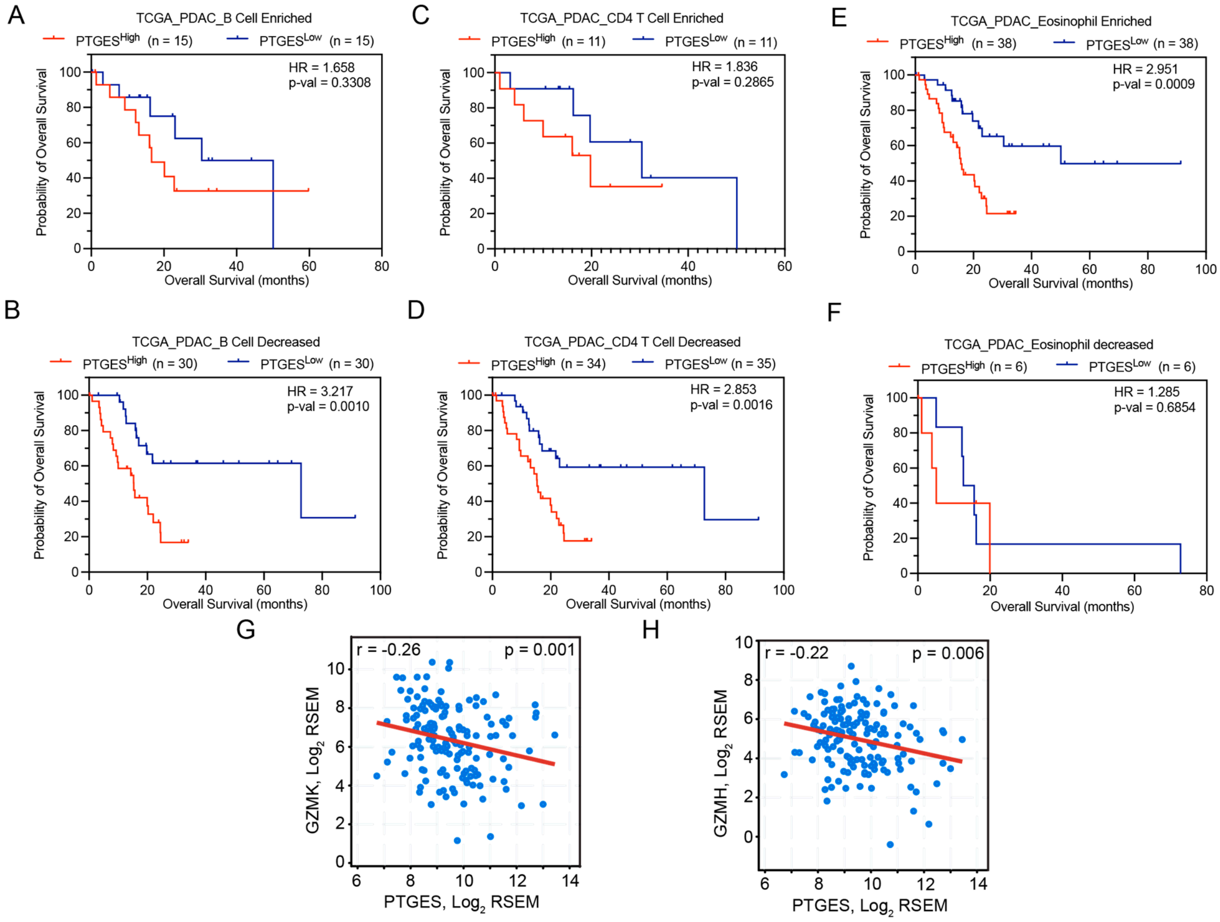

Figure S3. Correlation of immune population with PTGES expression in pancreatic cancer. (A & B) The Kaplan-Meier survival plots of overall survival differences between upper and lower quartiles of PTGES expression in PDAC patients with enriched (A) or decreased (B) B cell population. (C & D) The Kaplan-Meier survival plots of overall survival differences between upper and lower quartiles of PTGES expression in PDAC patients with enriched (C) or decreased (D) CD4+ T cell population. (E & F) The Kaplan-Meier survival plots of overall survival differences between upper and lower quartiles of PTGES expression in PDAC patients with enriched (E) or decreased (F) eosinophil population. (G & H) The spearman's rank correlation plots of marker genes (CD69, GZMK and GZMH) of activated in CD8+ T cells with PTGES expression in PDAC patients.
